# Dataset for assessing the scope and nature of global stream daylighting practices

**DOI:** 10.1016/j.dib.2020.106366

**Published:** 2020-10-06

**Authors:** Luna Khirfan, Niloofar Mohtat, Megan Peck, Andrew Chan, Lucas Ma

**Affiliations:** a School of Planning, University of Waterloo, ON, Canada; b City of Belleville, ON, Canada

**Keywords:** Stream daylighting, Deculverting, Nature-based solutions, Systematic literature review, Content analysis, Climate change

## Abstract

This paper presents five publicly available datasets (I through V) of which two are interactive and visual tools (a Tableau Dashboard and an Interactive Map). These five datasets were extracted from 115 literature sources on the daylighting of streams that were published between 1992 and 2018. *Dataset I* consist of 19 variables that combine two types of data extracted from these sources: ten manifest variables (indisputable, obvious, factual) and nine variables extracted from the sources’ latent content (indirect, hence, based on careful reading of the sources’ contents). Manifest variables include, among others, authors’ names and affiliations, authorship location, and publication year. Latent variables include primarily the literature sources’ underlying themes and their sub-themes (sub-categories), the daylighting case studies/projects discussed, and the geographic coverage or scope addressed in the literature sources. *Datasets II* identifies 16 literature sources that delve into the climate change adaptation and/or mitigation theme and reveal how it was tackled vis-à-vis the other themes/sub-themes. *Dataset III* identifies and provides detailed information on the 145 different stream daylighting case studies/projects mentioned in the literature's sources, such as each project's location, daylighted length, completion date, cost, and type of treatment. *Dataset IV* is a Tableau Dashboard that offers interactive analytical querying in the form of relational analyses and data visualization while *Dataset V* is an Interactive Map created in Google My Map that maps the 145 stream daylighting case studies/projects mentioned in the literature sources over and provides a synopsis on each based on the literature's contents. The combination of these five datasets and their diversity in type and presentation yields a comprehensive, global, and unique repository of information on the daylighting of urban streams for all types of audiences (academic, professional, and laypeople).

## Specifications Table

**Table:** 

Subject	Decision Sciences
Specific subject area	Urban Stream Daylighting
Type of data	Excel documents, Tableau Dashboard, Interactive Map, and tables, figure, and graphs
How data were acquired	All datasets were extracted from 115 literature sources (peer reviewed and grey literature) on stream daylighting using a combination of systematic literature review and content analysis. Datasets I, II, and III were recorded in Microsoft Excel documents. The ‘latent data’, in the form of notes extracted from the literature sources, were organized using Microsoft Word and EndNote then coded and transferred to Microsoft Excel documents. A primary key (PK) unique number was assigned to each literature source and ensured that these three datasets were connected. The computer software Alteryx and Tableau were used for data cleaning and data visualization/analytical querying respectively. Concurrently, Dataset III (dedicated to recording, from the literature sources’ latent content, details on the stream daylighting case studies/projects) were converted into CSV files and uploaded to a Google My Maps to create an Interactive Map of the stream daylighting projects around the world.
Data format	Mixed: raw and processed (summarized, filtered, and visual)
Parameters for data collection	The data collection includes four mandatory perimeters, namely: (1) Peer-reviewed and grey literature sources; (2) English language sources (with only one exception due to this source's relevancy and significance since it discusses the City of Zürich's (Switzerland) stream daylighting policy, which is unique in the world; (3) The literature sources were gleaned from Primo, Google Scholar, and Google search engines; and (4) The literature sources published between 1992 (when the first peer reviewed article on stream daylighting was published) and the end of December of 2018.
Description of data collection	The data were collected using four iterative steps, namely: (1) The identification of the relevant literature sources; (2) The characterization of the types of the included literature sources (e.g., peer reviewed articles, book chapters, government reports…etc.); (3) The assessment of the quality of the included literature sources; and (4) The evaluation of the search results
Data source location	Global data
Data accessibility	(1) Dataset I (The Stream Daylighting Literature Review) is publicly sharable are can be accessed and downloaded from the Mendeley Data Repository at: http://dx.doi.org/10.17632/j5zfp5vdz4.3 (2) Dataset II (The Stream Daylighting and Climate Change Literature Review Dataset) is publicly sharable are can be accessed and downloaded from the Mendeley Data Repository at: http://dx.doi.org/10.17632/j5zfp5vdz4.3 (3) Dataset III (The Stream Daylighting Literature Review Case Studies/Projects Dataset) is publicly sharable are can be accessed and downloaded from the Mendeley Data Repository: http://dx.doi.org/10.17632/dmpcs82b23.2 (4) Dataset IV (The Tableau Dashboard) is a publicly available interactive analytical querying (relational analyses) tool that can be viewed/engaged with through: https://uwaterloo.ca/stream-daylighting/literature-review-database and https://public.tableau.com/profile/nathan.woodcock5796#!/ (5) Dataset V (The Interactive Map) is a publicly available interactive spatial tool that can be viewed/engaged with at: https://uwaterloo.ca/stream-daylighting/interactive-map (note: the research team welcomes contributions to the Interactive Map)
Related research article	10.1016/j.wasec.2020.100067 Khirfan, L.; Mohtat, N.; Peck, M.; A Systematic Literature Review and Content Analysis Combination to “Shed Some Light” on Stream Daylighting (Deculverting), *Water Security*, June 2020 10.1016/j.scs.2020.102225 Khirfan, L.; Peck, M.; Mohtat, N.; Digging for the truth: A combined method to analyze the literature on stream daylighting, Sustainable Cities and Society, Volume 59, August 2020, 102,225

## Value of the Data

• These datasets provide an unprecedented repository of information on stream daylighting, including, among others, the literature's contents, the stream daylighting case studies/projects, and the disciplines and publishers focusing on stream daylighting. They therefore constitute a reference point for anyone (academics, practitioners, and laypeople) interested in the daylighting/deculverting of streams.
• The comprehensiveness of the datasets (systematic literature review) and their diversity in nature (Excel documents, Tableau dashboard, and interactive maps) and in content (multiple variables, the literature's manifest and latent content, and the daylighting case studies/projects) render them beneficial for further research for various academic disciplines and fields of practice (e.g., urban planning and design, water resource engineering, ecology, and climate change to name but a few).
• These datasets may be mined by researchers and practitioners for further analyses to facilitate a better understanding of both the scope and the nature of the stream daylighting literature and its practice around the globe.
• These datasets provide a springboard for future (longitudinal) reviews of the literature on stream daylighting.
• The diversity of the types of these datasets (Excel documents, Tableau dashboard, and interactive maps) renders them ideal for use by educators for training on data mining, statistical analyses, and representation.

## 1 Data Description

*Dataset I (The Stream Daylighting Literature Review):*

Dataset I exist in the form of an Excel document with 19 variables that spread over seven separate sheets whereby these sheets are united by a unique identifier, a “Primary Key” (PK) number, for each literature source –in other words, each source's unique PK is used across all these seven sheets (and also, across the other Datasets II and III). Table 1 lists the 19 variables, their location in Dataset I (sheet and column), and a description of each variable [1]. Notably, the combination of variables that fall under ‘manifest data’ constitute this study's sources –in other words, the list of all the 115 literature sources gathered on stream daylighting that were used as a bases for this research project.

**Table 1:** Explanation of the contents of the Microsoft Excel document that constitutes Dataset I.

		Location in the dataset		
Data type	Variable	Sheet title	Column number	Description
**PK**	Primary Key (PK)	All sheets	A	The PK is a unique number for each literature source. In Dataset I, the PK number occupies Column A of every one of the seven sheets. Also, we included the PK number in Datasets II and III.

**Manifest Data**	Authors’ names	Datasheet	B	The names of the literature sources’ authors
	Publication year	Datasheet	C	The year the literature source was published
	Source's title	Datasheet	D	The full title of the literature source
	Journal/Publisher	Datasheet	E	The name of the journal and/or the publishing institution
	Peer reviewed (Yes/No)	Datasheet	F	Whether or not the literature source is peer reviewed
	Daylighting case study mentioned	Datasheet	G	Whether or not the literature source mentions a daylighting case study/project
	Publication origin location	(a) Publication origin	B	The authorship's geographic location by city or state or province
	Publication origin country	(a) Publication origin	B	The authorship's geographic location by country
	Publication origin continent	(a) Publication origin	C	The authorship's geographic location by continent
	Publication type	(b) Publication Type	B	Seven types of literature sources were identified: journal article, book, book chapter, institutional report, conference paper, supervised student work (Master's or Doctoral research), and workshop proceedings

**Latent Data**	Theme	(c) Theme	B	The main theme on stream daylighting discussed in the literature source
	Sub-Theme	(d) Sub-Theme	B	The sub-theme(s) on stream daylighting discussed in the literature source
	Case study	(e) Case Study Names + Location	B	The name of the stream daylighting case study/project
	Case Study Location	(e) Case Study Names + Location	C	The daylighting case study/project location by city or state or province
	Case Study Country	(e) Case Study Names + Location	D	The daylighting case study/project location by country
	Case Study Continent	(e) Case Study Names + Location	E	The daylighting case study/project location by continent
	Geographical Scope Location	(f) Geographical Scope	B	The geographical areas discussed in the literature source by state or province or country
	Geographical Scope Country	(f) Geographical Scope	C	The geographical areas discussed in the literature source by country
	Geographical Scope Continent	(f) Geographical Scope	D	The geographical areas discussed in the literature source by continent

**Fig. 1:**
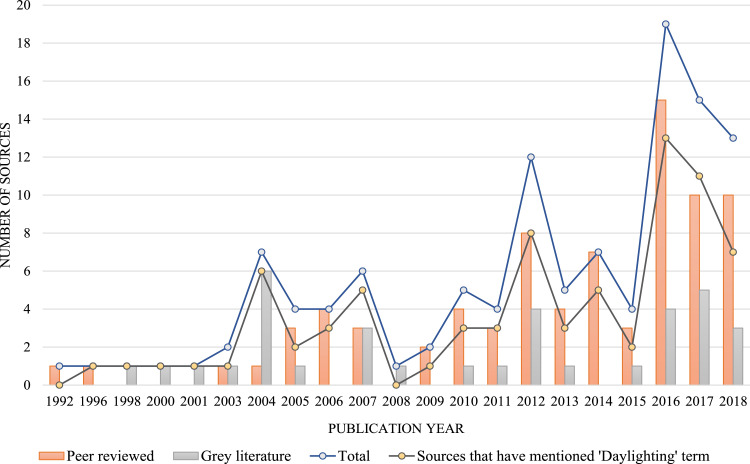
The distribution of stream daylighting publications over time.

**Fig. 2:**
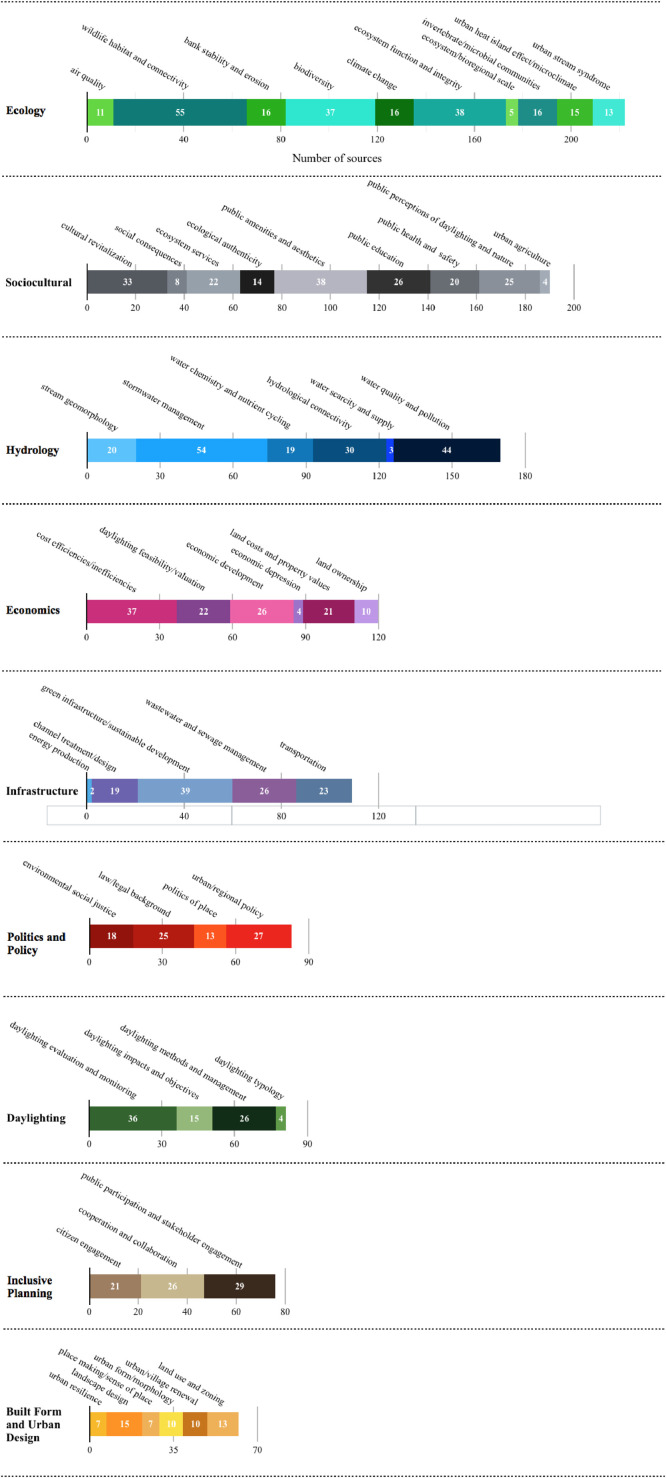
The prevalence of literature's sub-themes (i.e., the number of articles categorized under each subtheme).

**Fig. 3:**
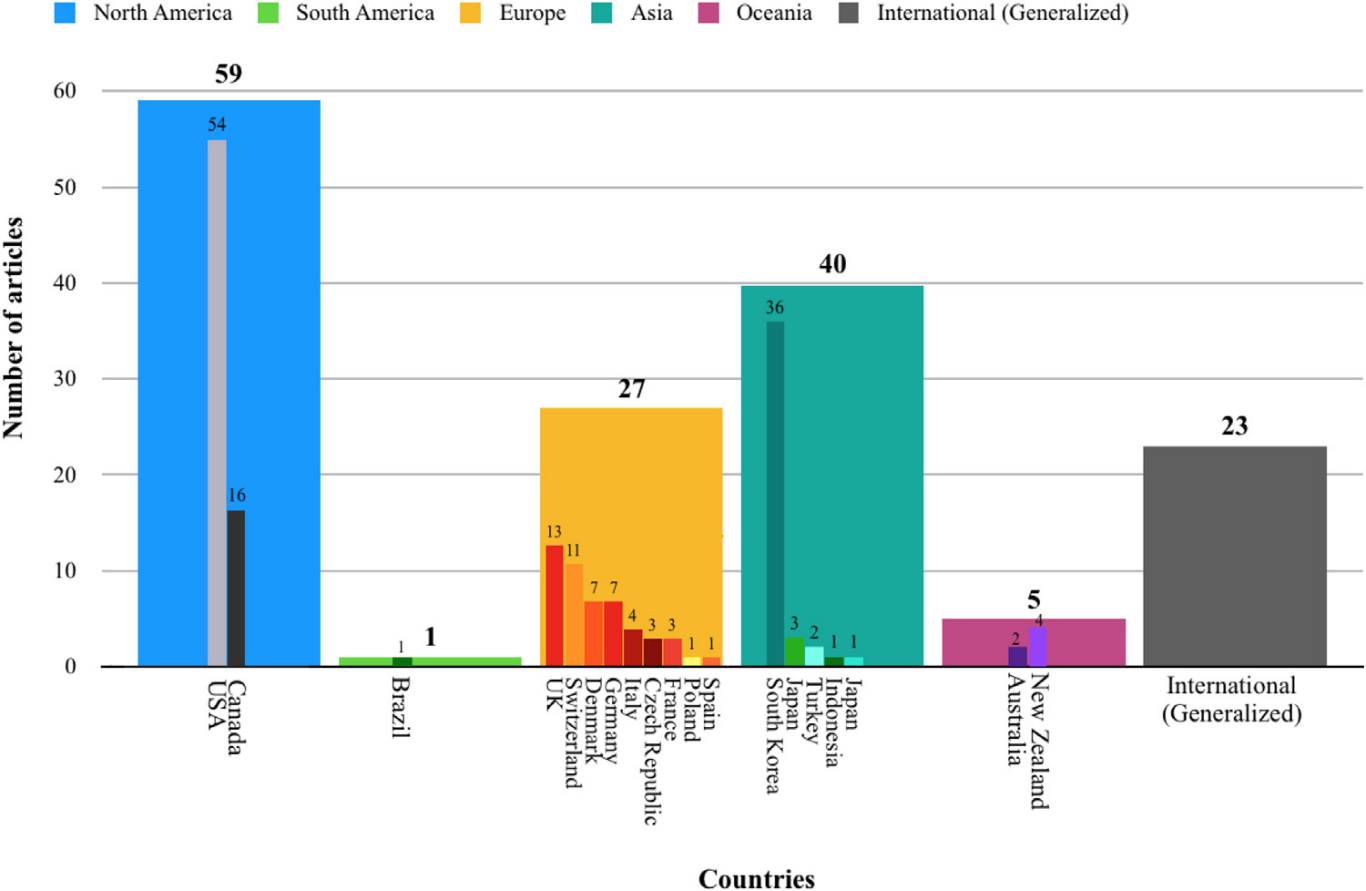
The literature's geographical scope by continent and country. The categories are non-exclusive, meaning one article can have more than one geographic scope; therefore, the sources’ counts do not sum to 115 (the total of literature sources).

**Fig. 4:**
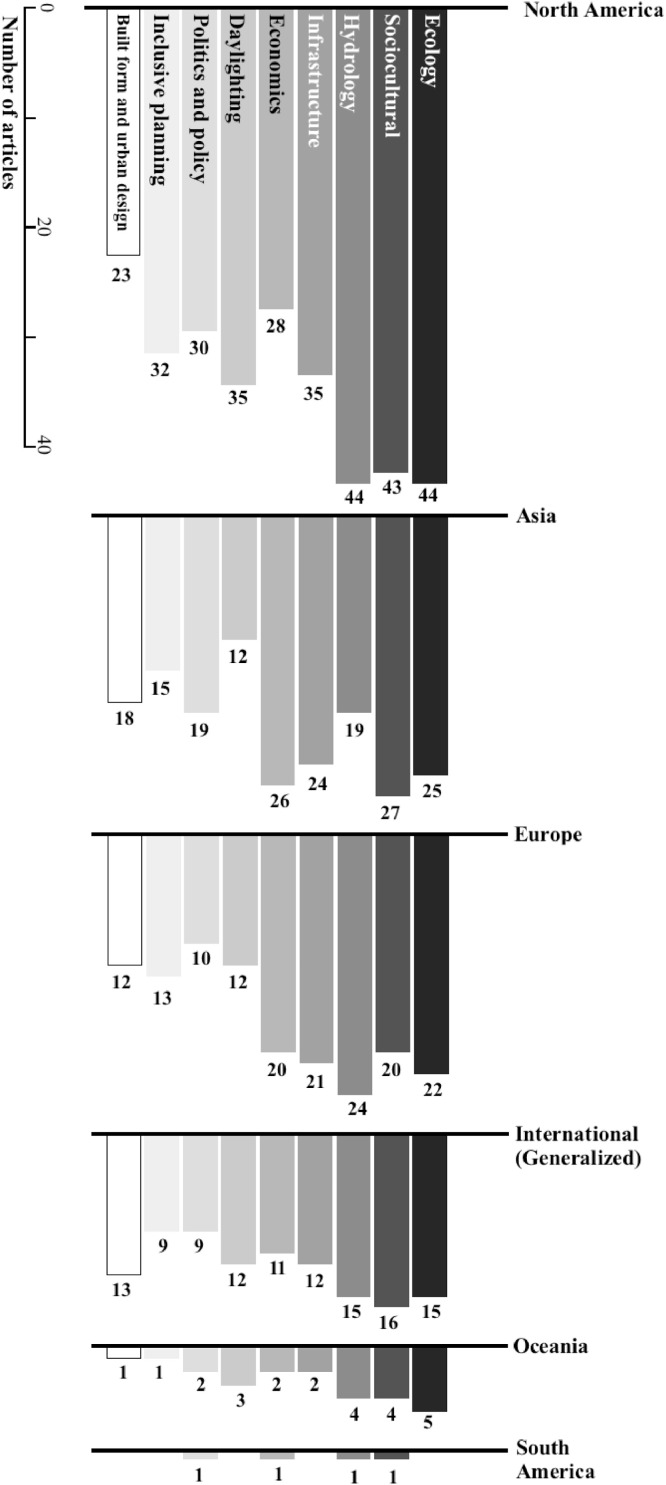
The connections between the frequency of stream daylighting themes and the geographic scope in the literature's discussions of stream daylighting.

We extracted Dataset I's 19 variables from a total of 115 literature sources on stream daylighting. Of these variables, 10 are manifest data (the literature's obvious and indisputable attributes), such as: authors’ names and affiliations, title and year of the source's publication (see [2] for detailed analyses of these manifest variables). For example, Fig. 1 shows the distribution of stream daylighting publications over time. The remaining nine variables are latent data (extracted from the sources’ content through careful reading and codified notes). These nine latent data variables cover four underlying subject matters discussed in the literature sources, namely: 1) the higher-order themes (we identified a total of nine); 2) the second-order categories or sub-themes (we identified a total of 53 – Fig. 2 overlays the number of sources that discussed each of these sub-themes); 3) the daylighting case studies/projects (we identified 145 which led to an expanded Dataset III); and 4) the geographic coverage or scope addressed in the literature sources (in the absence of a clear geographic focus -whether continent and/or country- we coded the source as “international (generalized)”) [3]. The geographic scope of the source's discussion is a non-exclusive characteristic, meaning a single publication can have more than one geographic focus. Of this study's 115 literature sources, 51%, 35%, 23%, 20%, 4% and 0.87% are contextually focused on a North American, Asian, European, international, Oceanic and, South American scopes, respectively (Fig. 3). We also connect the nine higher-order themes and the geographic scope in the literature's discussions of stream daylighting (see Fig. 4) [1].

Notes:

(1) In addition to providing the foundation for Datasets II and III, this Dataset I served as the backbone for the research project's dashboard produced in Tableau (Dataset IV)
(2) The PK number, each source's unique number, connects Datasets I, II, and III. In other words, the PK number is used in Datasets II and III to identify the literature sources that discuss stream daylighting vis-à-vis climate change and those that specifically mention a particular stream daylighting case study/project.

*Dataset II (The Stream Daylighting and Climate Change Literature Review Dataset):*

**Fig. 5:**
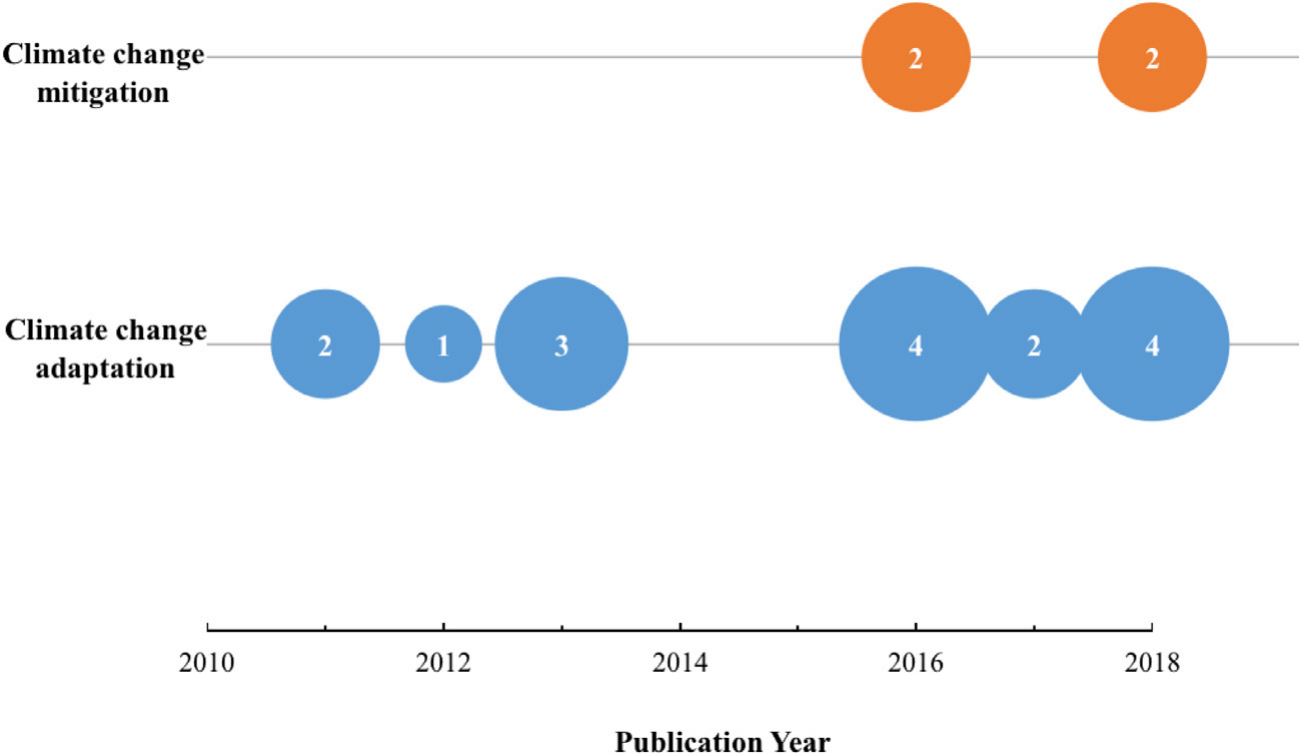
The number of stream daylighting literature sources that have (directly and/or indirectly) referred to climate change adaptation and/or mitigation per publication year.

**Fig. 6:**
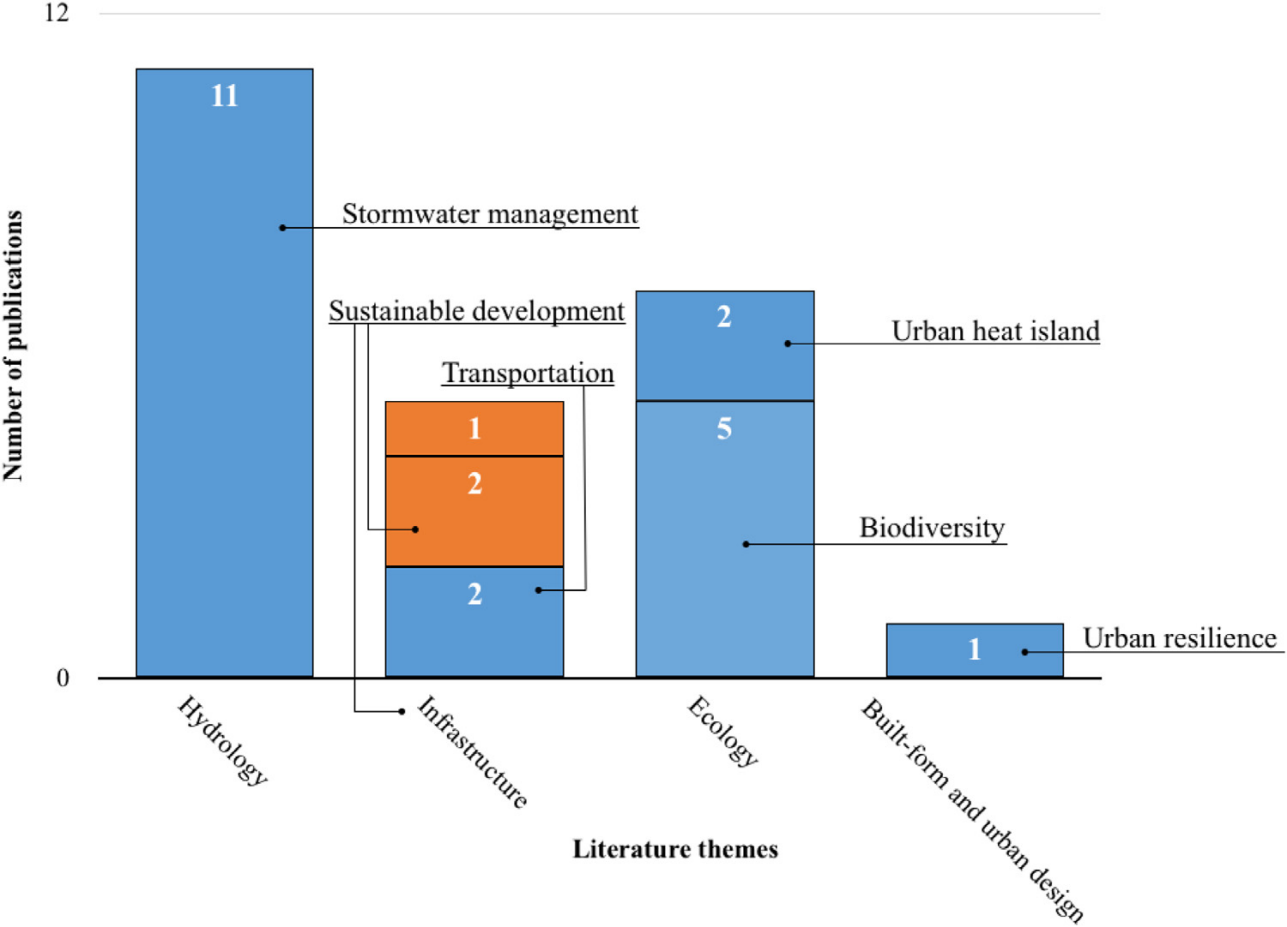
The stream daylighting literature sources that discuss climate change adaptation and mitigation and their overlapping literature themes (each source may address one or more theme or subtheme).

This Dataset II is in the form of a Microsoft Excel document organized over nine columns. Dataset II delves deeper into one of the latent content variables extracted from the literature's sources namely, the climate change theme [3]. In Dataset II, we identify 16 literature sources on stream daylighting that also discuss (directly or indirectly) climate change. Notably, these 16 sources were all published after 2010 (Fig. 5). We sought from these sources information on stream daylighting as a nature-based solution in the age of climate change, specifically: 1) how do these sources address stream daylighting in relation to climate change? 2) Do these sources tackle climate adaptation, mitigation, or both? and 3) how do these literature sources tackle stream daylighting and climate adaptation and/or mitigation vis-à-vis the nine higher-order themes and the 53 sub-categories (sub-themes)? For example, these 16 sources discuss climate change adaptation and mitigation in relation to four of the literature's subthemes, namely (in descending order): hydrology, infrastructure, ecology, and built form and urban design (see Fig. 6 and refer to Dataset II through [1]).

*Dataset III (The Stream Daylighting Literature Review Case Studies/Projects Dataset):*

This *dataset* includes the latent data extracted from the literature sources on the stream daylighting case studies/projects. Accordingly, we identify a total of 145 different stream daylighting case studies/projects that we organize in a Microsoft Excel Document in which we specify 20 variables that are explained in Table 2. Similar to the case studies/projects, we primarily extracted these variables from the latent contents of the 115 sources of literature on the daylighting (i.e., deculverting) of streams, however, we augmented this Dataset III with other sources (listed under column number T in this dataset) (see [4]). These variables offer detailed information of the stream daylighting case studies/projects including: the names of the daylighting project and of the daylighted/deculverted stream or river; locational information (including, coordinates); completion year; treatment type; cost information (including, cost per meter); innovative implementation techniques (if any); and even whether the daylighted/deculverted stream is visible in Google Maps.

**Table 2:** Explanation of the variables in the Microsoft Excel document that constitutes Dataset III.

Column number	Label	Explanation
A	AB Ref #	A unique number for each case study/project
B	Project Name	The project's name
C	Stream/River	The stream's/river's name
D	Town/City	The town or city where the case study/project is located
E	Region/Province/State	The region, province, or state where the case study/project is located
F	Country	The country where the case study/project is located
G	Completion Year	The year the stream daylighting project was completed
H	Treatment Type	The type of stream daylighting treatment, whether “naturalized” river morphologythat mimics pre-culverting conditions or “channelized” within a hard-engineered infrastructure (e.g., concrete, stone, or brick).
I	Length (meters)	The length (in meters) of the daylighted segment of the stream/river
J	Cost (US $)	The total cost of the stream daylighting project
K	Cost Year	The year in which the total cost of the stream daylighting project was calculated
L	Cost per metre (unadjusted)	The cost per meter during the year in which the total cost was calculated
M	Cost per metre	The cost per meter adjusted to rates in 2018
N	Innovation(New techniques or approaches) in the execution	The new and innovative techniques or approaches adopted in the project
O	First Coordinate	The location coordinates of the daylighted stream
P	Second Coordinate	
Q	Extent of the location's visibility on Google Maps	How visible is the case study's/project's location on Google Maps
R	Related Literature Sources	The literature sources that discuss this case study/project
S	PK	The PK (Primary Key number) for each of the literature sources that discuss this case study/project
T	Notes on the sources of information on the case study/project	Any additional notes

**Fig. 7:**
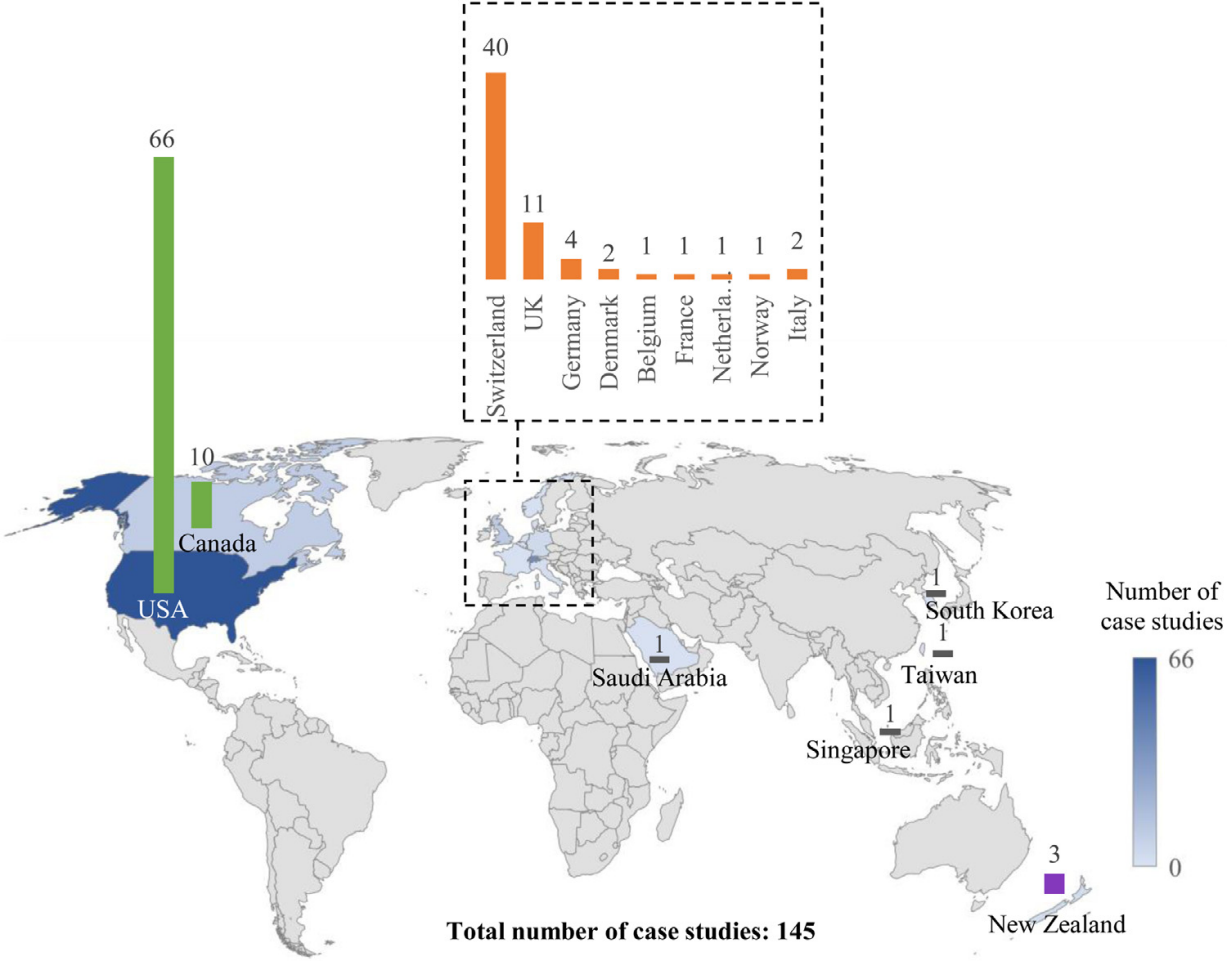
The global distribution of the case studies mentioned in the literature.

The 145 stream daylighting case studies/projects mentioned in the 115 literature sources spread across four continents (North America, Europe, Asia and Oceania) a9nd 16 countries. The global distribution of these case studies, and the number of case studies located in each continent and country are seen in Fig. 7.

**Fig. 8:**
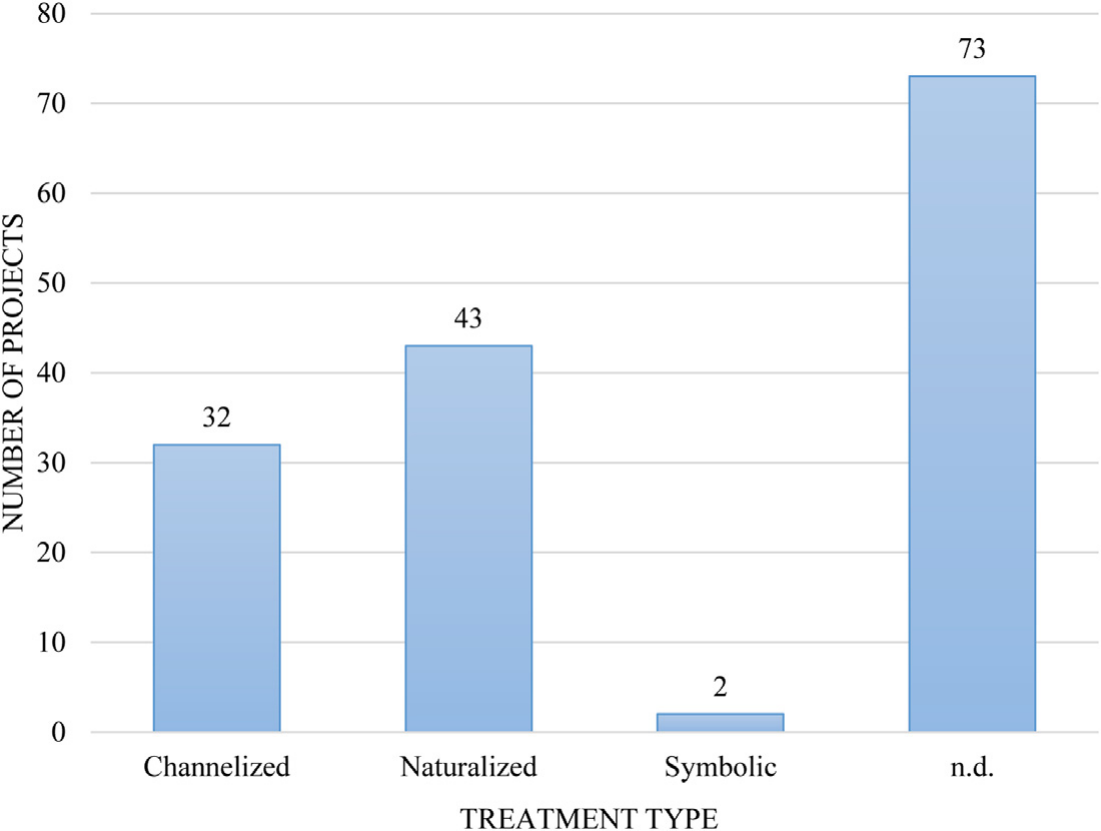
Daylighting projects by treatment type.

Our data also reveal the treatment type of the 145 stream daylighting case studies/projects –i.e., whether the treatment of the daylighted streams adopts a naturalized morphology that mimics their pre-culverting conditions or channelized within a hard-engineered infrastructure (e.g., concrete, stone, or brick). Generally, the literature sources do not identify the treatment type. Indeed, our Database III reveals that the largest proportion of daylighted streams had an unidentified treatment type (indicated as ‘n.d.’ in Database III) in the literature (73 of 145) –in other words, the source's text does not allow us to determine whether these streams were naturalized or channelized. Of those that were identified, more projects were classified as naturalized (43 of 145) than as channelized (32 of 145), indicating that there is a greater record of naturalized daylighting systems than ones using hard-engineered infrastructure. Two projects were identified as ‘symbolic’, rendering them the exception in terms of treatment (Fig. 8) [4].

**Fig. 9:**
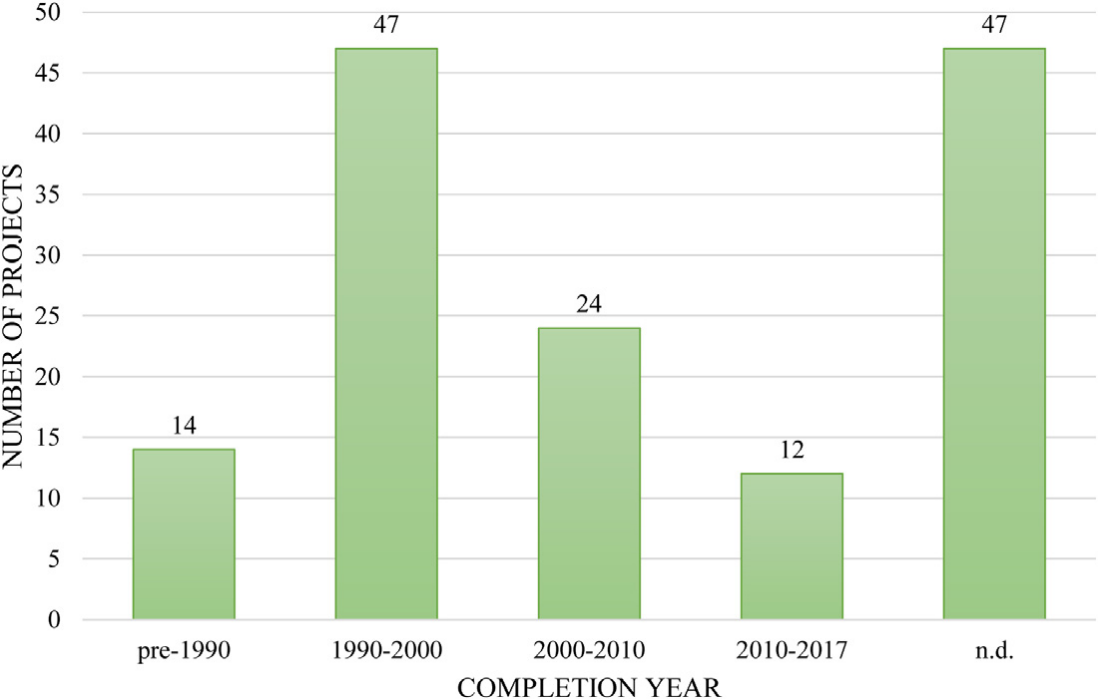
Daylighting projects by completion year (*n* = 145).

Database III also gives insights into the uptake of the stream daylighting practice over time (Fig. 9). Indeed, the decade between 1990 and 2000 witnessed the largest number of completed stream daylighting projects (47 of 145). Between 2000 and 2010 a total of 24 streams were daylighted, while those whose daylighting precedes 1990 totaled 14 streams and the ones daylighted during the 2010s total 12. For 47 of the 145 projects, a completion date could not be identified from the literature sources [4].

**Fig. 10:**
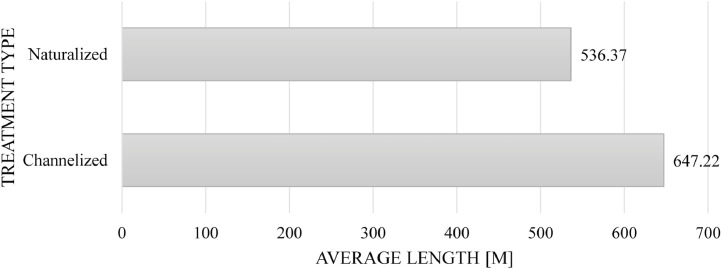
The treatment type and average length of daylighting projects (*n* = 62).

Our Database III also gives insights into the length of the daylighted segments of the streams as well as the cost of daylighted per meter. Table 3 and Fig. 10 summarize and display the gathered data whereby of the 32 channelized daylighted streams, we found the data on the total length for 24 streams, while of the 43 naturalized daylighted streams, we found the data on the total length for 38 streams.^1^ There does not seem to be a strong correlation differentiating these treatment types, as individual projects in both categories are scattered around a similar range (i.e., anywhere from 100 m to 1000 m). Instead, this discrepancy is attributed to a single case of the Cheonggyecheon, whereby as the longest stream (at 5800 m), it skews the average length for the ‘channelized’ category upward [4].

**Table 3:** A breakdown of the daylighted streams by treatment type (channelized versus naturalized) as gleaned from the literature sources.

Treatment type	Average length (m)	Total length (m)	Count (excluding n.d.)
Channelized	647.22	15,533.44	24 of 32
Naturalized	536.37	20,382.32	38 of 43

**Fig. 11:**
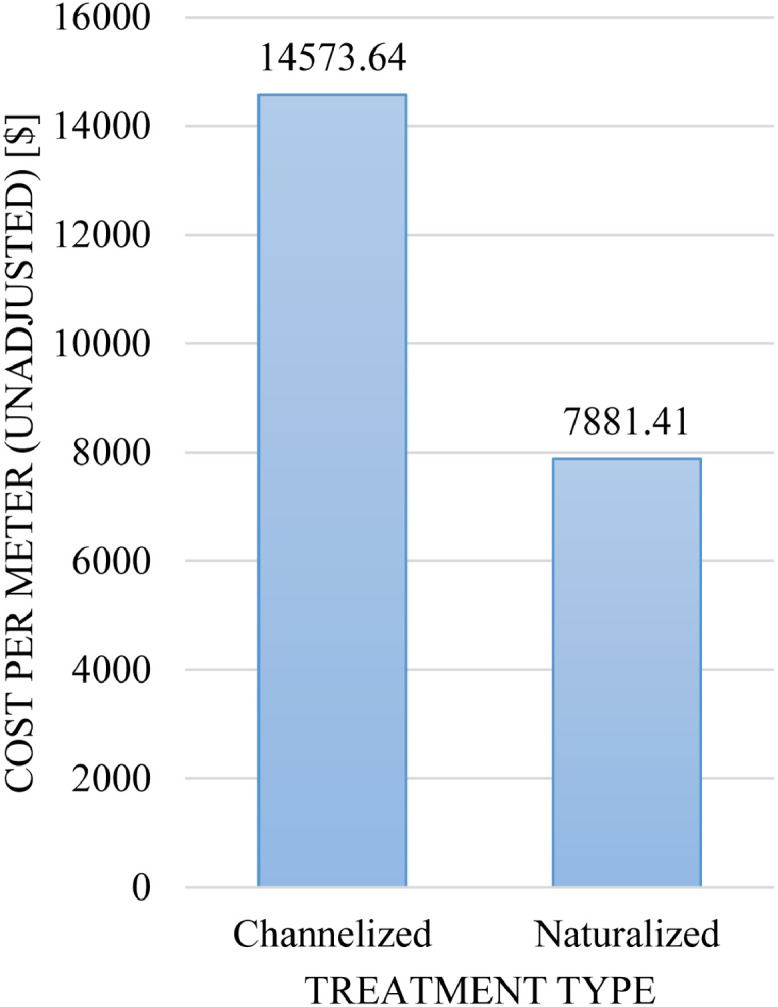
Average cost per meter (unadjusted) of daylighting projects by treatment type (*n* = 32).

Database III also gives insights into the cost per daylighted meter. Information on the cost of stream daylighting was, for the most part, lacking in the literature sources. Considering the relatively small sample size,^2^ Table 4 and Fig. 11 provide insights on the average cost per meter for each treatment type. The data reveal that the channelized treatment is far more costly than the naturalized treatment. Indeed, the deviation is significant between the average costs for each type, from less than $100 per meter for the naturalized treatment to several thousands of dollars per meter for the channelized treatment. A probable explanation for the higher cost of the channelized treatment is that it involves the construction of and investment in additional infrastructure (e.g., transit options as is the case for the Cheonggyecheon) [4].

**Table 4:** The average cost per meter for each treatment type of stream daylighting as extracted from the literature sources.

Treatment type	Average cost per meter (unadjusted)	Total cost per meter (unadjusted)	Count (excluding n.d.)
Channelized	14,573.64	116,589.15	8 of 33
Naturalized	7881.41	197,035.3512	24 of 43

**Fig. 12:**
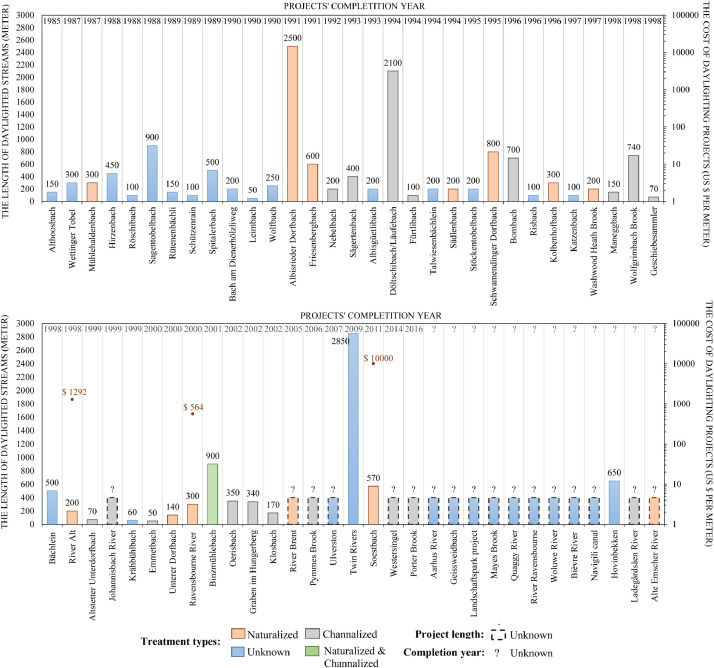
An overlay of some of the data available in the literature sources on the stream daylighting case studies/projects in Europe (cost/meter, length, completion year, and treatment type).

**Fig. 13:**
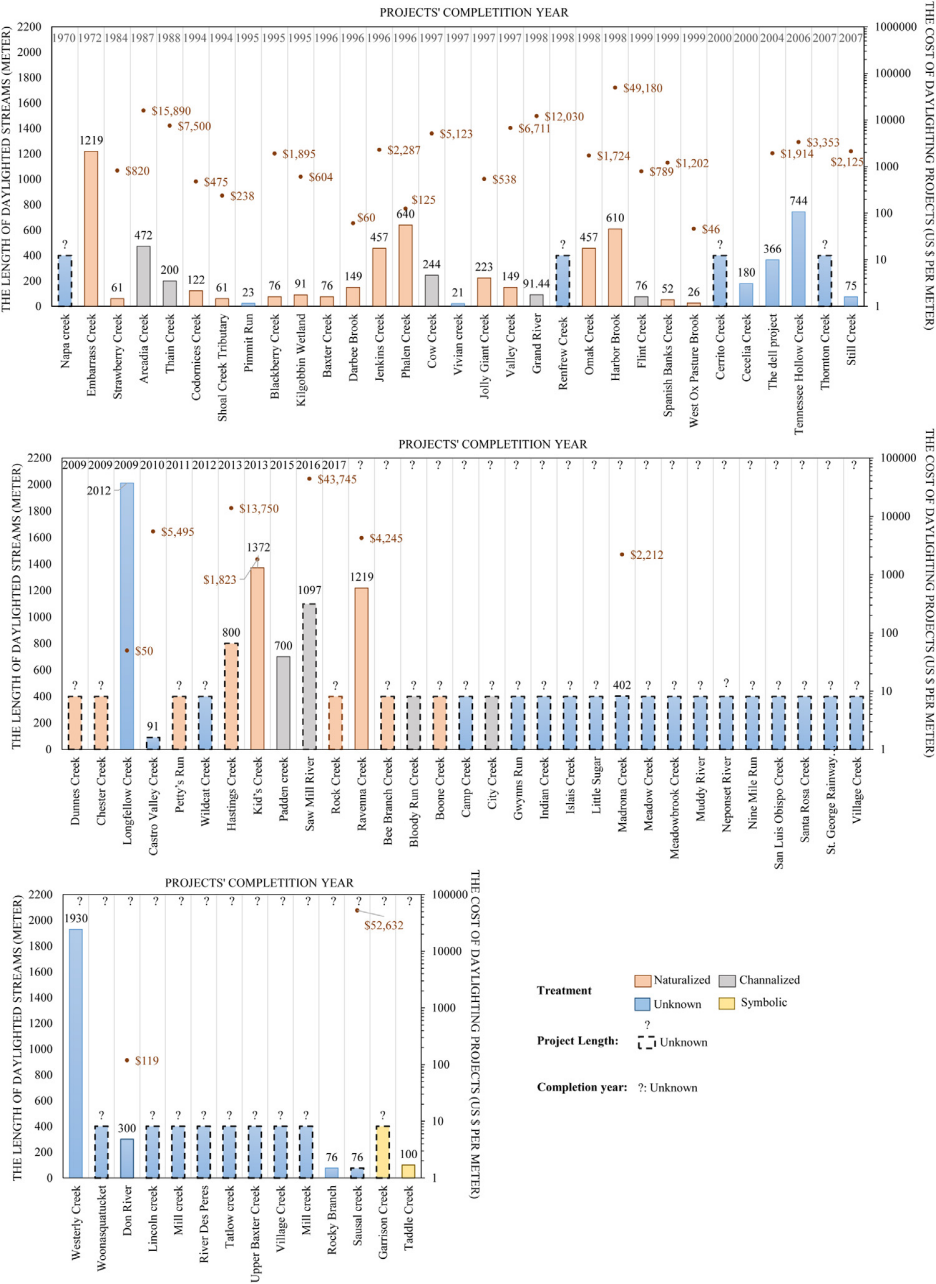
An overlay of some of the data available in the literature sources on the stream daylighting case studies/projects in North America (cost/meter, length, completion year, and treatment type).

**Fig. 14:**
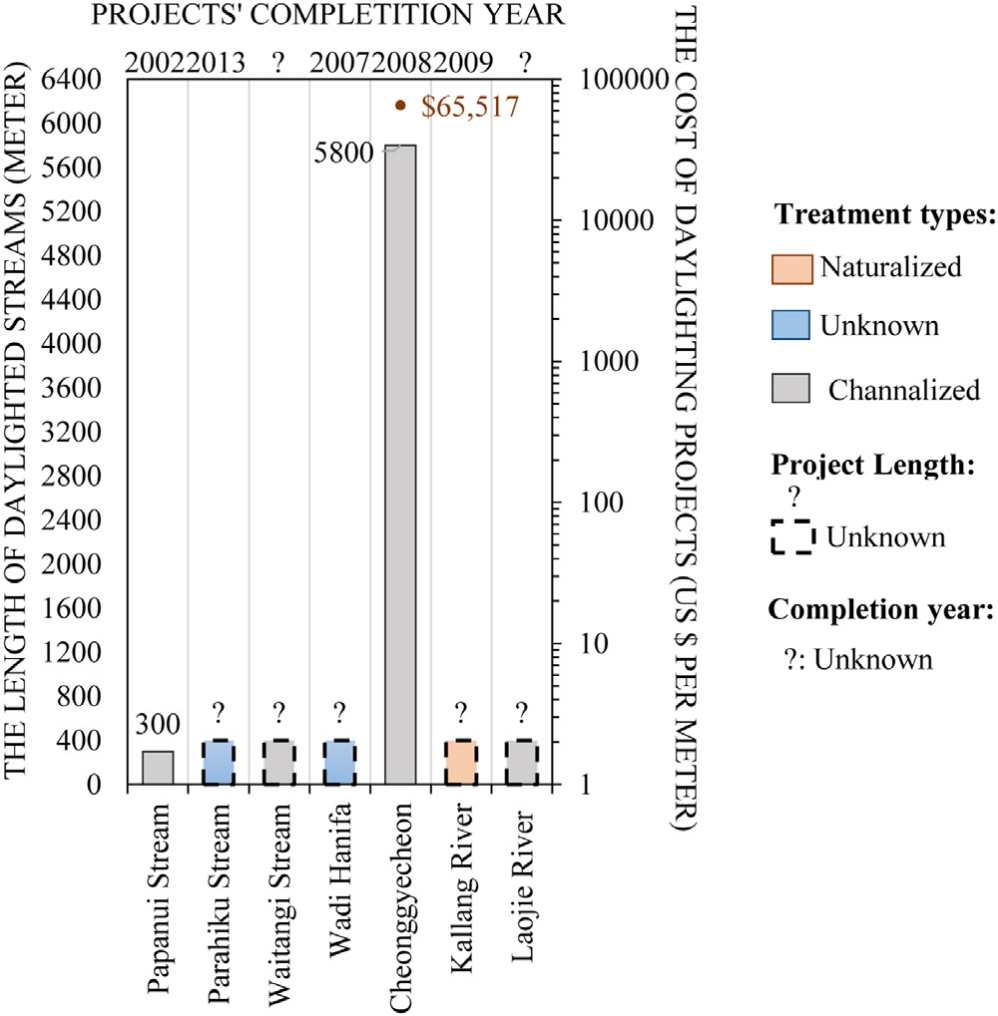
An overlay of some of the data available in the literature sources on the stream daylighting case studies/projects in Oceania and Asia (cost/meter, length, completion year, and treatment type).

**Fig. 15:**
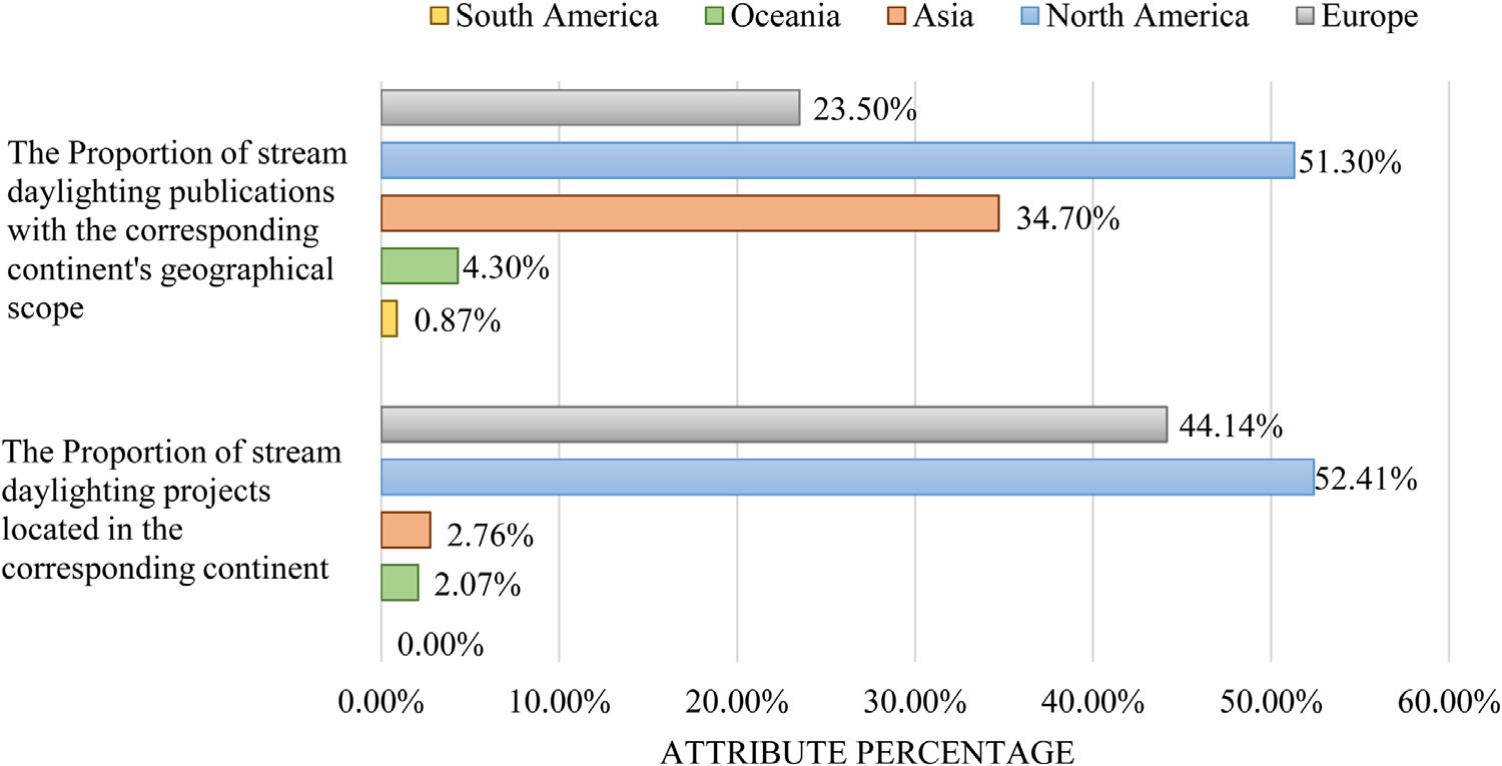
The number of stream daylighting projects, per geographic location, versus the number of publications with the corresponding geographic scope.

In Fig. 12, Fig. 13, Fig. 14, we connect the geographic location of the stream daylighting case studies/projects with other variables (completion year, treatment type, daylighted length, and cost per meter). The most frequently mentioned case study/project in the literature is the Cheonggyecheon^3^ stream restoration project in Seoul, South Korea, followed first by Strawberry Creek (California, USA), and second by Blackberry Creek (California, USA). Table 5 shows the stream daylighting literature's 26 most frequently cited case studies and their corresponding geographic locations while the relationship between the proportion of stream daylighting case studies and the literature's geographical scope/focus is featured in Fig. 15 [4].

**Table 5:** The 26 most frequently cited (three times and more) stream daylighting case studies/projects in the literature and their geographic locations.

		Case study location	
Case study	Number of articles	City/State	Country
Cheonggyecheon Stream	44	Gyeonggi-do	South Korea
Strawberry Creek	14	California	USA
Blackberry Creek	9	California	USA
Arcadia Creek	7	Michigan and Mississippi	USA
Codornices Creek (California, USA)	6	California	USA
Jolly Giant Creek	6	California	USA
Phalen Creek	6	Minnesota	USA
Albisrieder Dorfbach	5	Zurich	Switzerland
Baxter Creek	5	California	USA
Cow Creek	5	Kensas	USA
Darbee Brook	5	New York	USA
Spanish Banks Creek	5	British Columbia	Canada
Thornton Creek	5	Washington	USA
Valley Creek	5	Washington	USA
Embarrass Creek	4	Illinois	USA
Jenkins Creek	4	Washington	USA
River Alt (Merseyside, England)	4	Merseyside	United Kingdom
West Ox Pasture Brook	4	Massachusetts	USA
Napa Creek	3	California	USA
Nebelbach	3	Zurich	Switzerland
Omak Creek	3	Washington	USA
Pimmit Run Tributary	3	Virginia	USA
Saw Mill River	3	New York	USA
Shoal Creek Tributary	3	Georgia	USA
Taddle Creek	3	Ontario	Canada
Thain Creek	3	British Columbia	Canada

Notes:

(1) We use the same primary key number (PK) to connect this Dataset III to our other two Datasets I and II.
(2) It is imperative to stress that the literature sources constituted the primary source of data for this Dataset III. In other words, while there are other stream daylighting projects around the world, this dataset is limited to those mentioned in the literature sources (peer reviewed and grey literature).

*Dataset IV (The Tableau Dashboard):* The Microsoft Excel document of Dataset I, which was cleaned using the software Alteryx, provided the analytical backbone for the Tableau Dashboard. The use of Tableau streamlined analytical querying, specifically relational analyses and aided in the visualization of data trends. Dataset IV (the Tableau Dashboard) is publicly available, in the form of an interactive tool, through the following links:

https://uwaterloo.ca/stream-daylighting/literature-review-database
and/or
https://public.tableau.com/profile/nathan.woodcock5796#!/

**Fig. 16:**
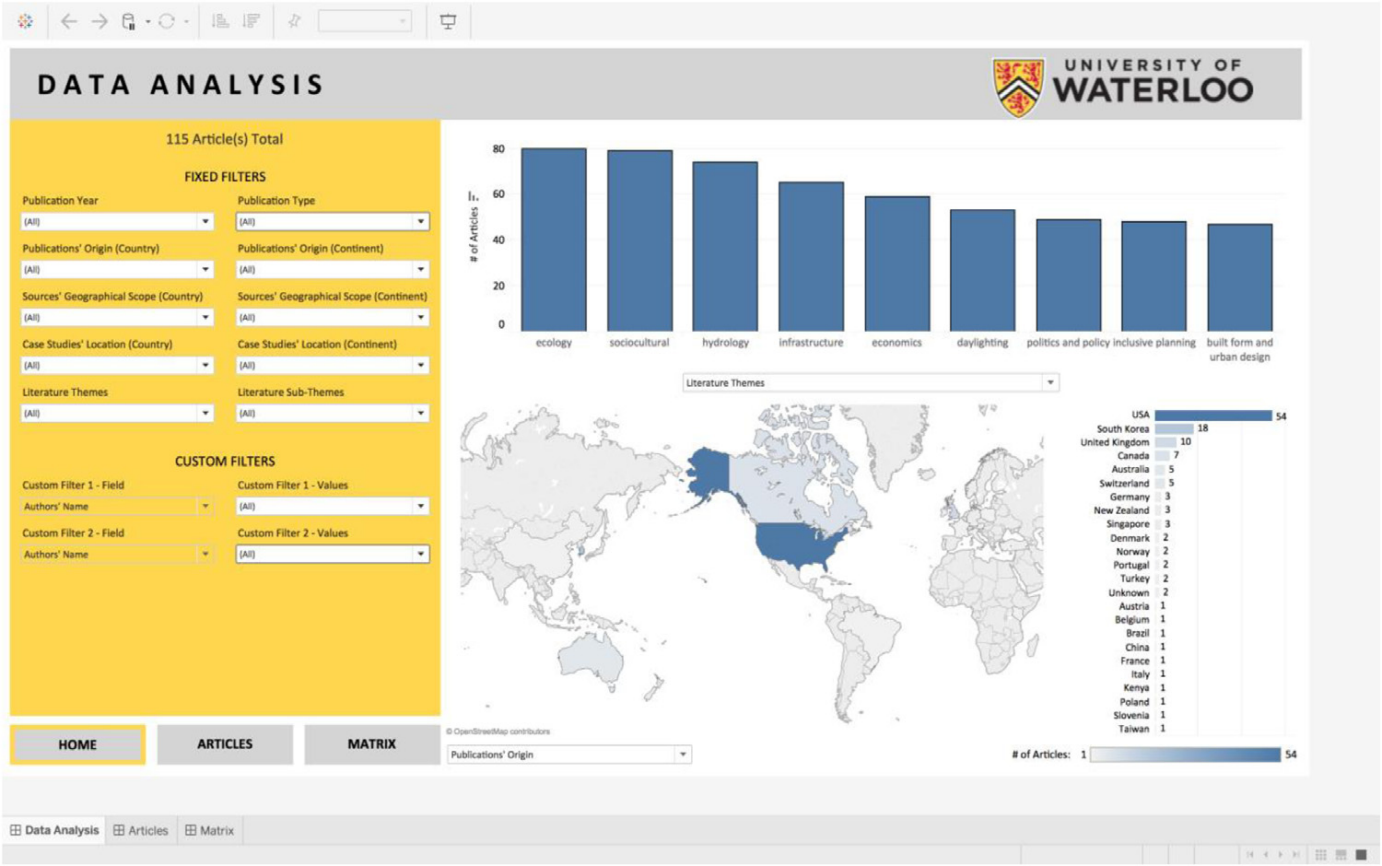
The Tableau dashboard's interface.

Visitors to the research project's website can engage with the data by generating their own analytical queries in the form of relational analyses and data visualization –in other words, it is an interactive visual manifestation of Dataset I (Fig. 16).

*Dataset V (The Interactive Map):* The Microsoft Excel document of Dataset III provided the backbone for a publicly available Interactive Map created in Google My Maps and available through:

https://uwaterloo.ca/stream-daylighting/interactive-map.

The Interactive Map allows site visitors to engage with it in various ways whereby they can explore the various stream daylighting case studies/projects that are mentioned in the literature sources. Website visitors can also share their input via a dedicated email address: streamdaylighting@uwaterloo.ca

**Fig. 17:**
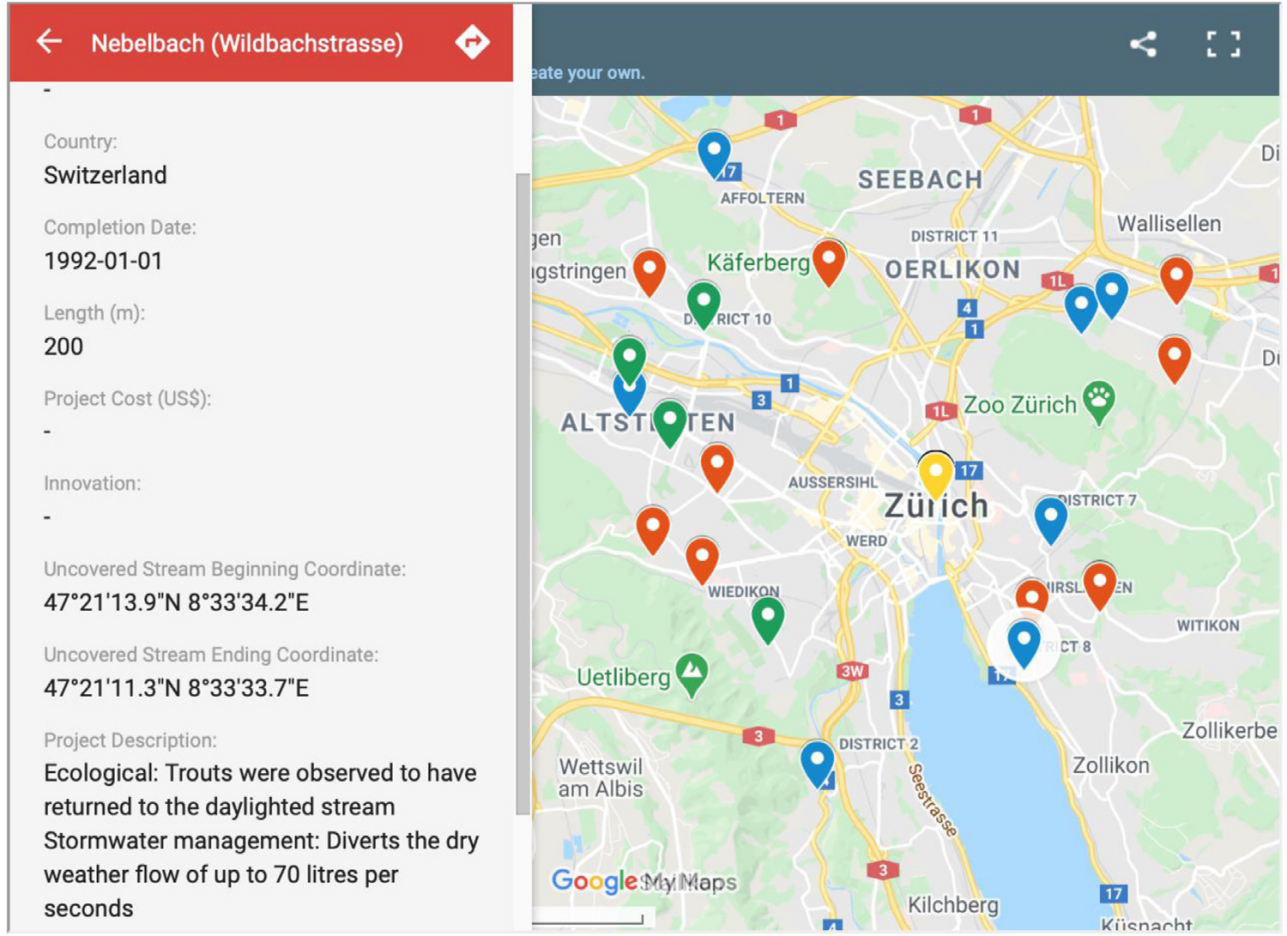
The Interactive Map's interface.

The interface also allows visitors to distinguish between the various types of stream daylighting treatments –that is, whether the daylighted stream is channelized or naturalized and provides information, extracted and compiled from the literature sources, on the case studies/projects (Fig. 17).

## 2 Experimental Design, Materials and Methods

Datasets I through V ensued from a systematic review of 115 English language literature sources on the daylighting/deculverting of streams (with one exception: a Swiss-German Report on the City of Zürich's unique daylighting policy). We combined this systematic review, which uncovered the manifest data, with content analysis to extract from these sources their latent content, in reference to the literature sources’ less obvious characteristics and information that can be uncovered only by a thorough reading of each source's text [5].

**Fig. 18:**
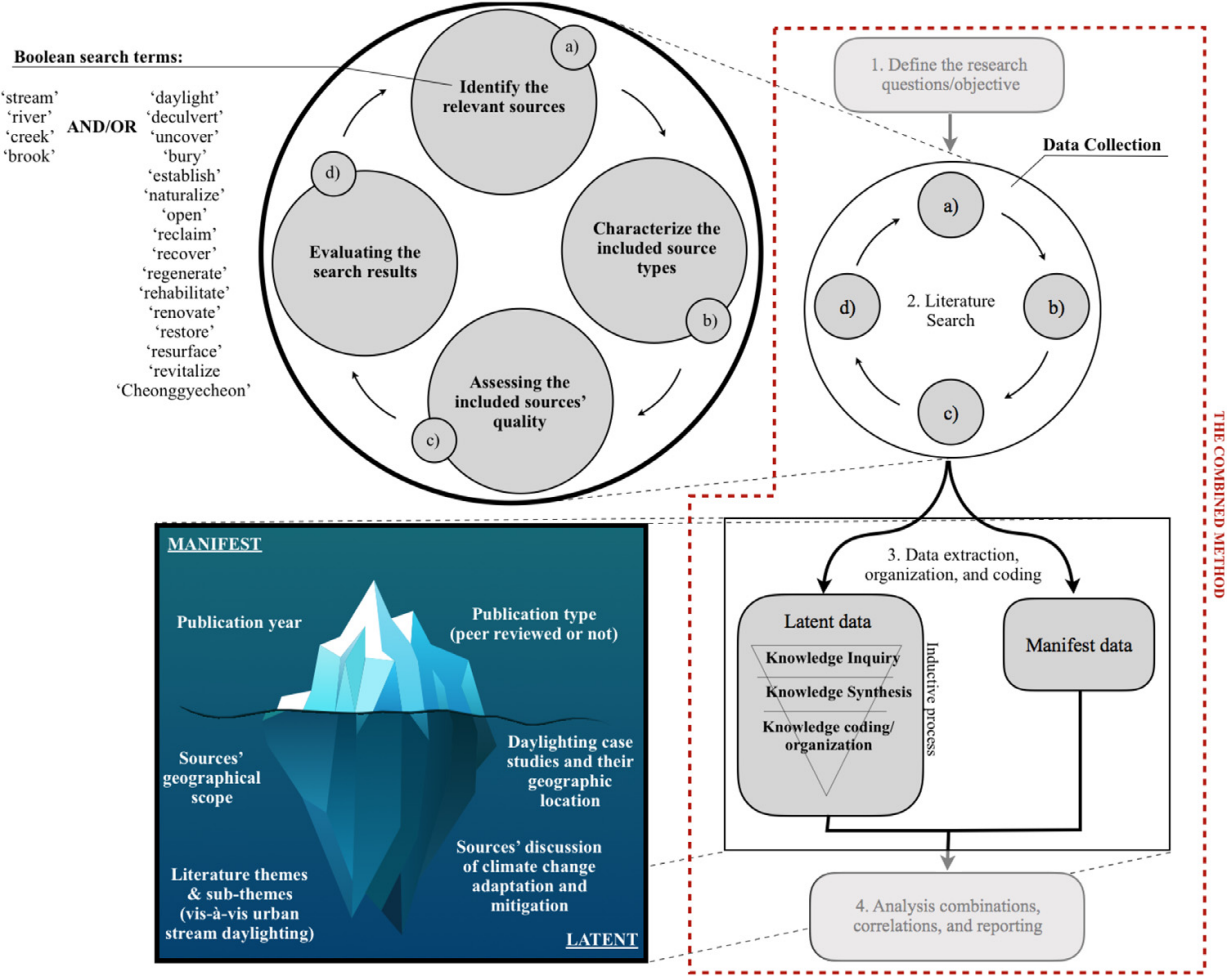
The combined method highlighting the process for collecting manifest and latent data.

Our combination of systematic literature review and content analysis facilitated a better understanding of the temporal and thematic evolution of the discourse on stream daylighting and the current status of the empirical studies on the subject. Specifically, by including all the sources tackling stream daylighting, the systematic literature review facilitated an understanding of the literature's general scope and nature. Furthermore, by integrating content analysis into our methods, we ensured a critical review that derived rich meanings from the data while providing methodological structure that reduced research bias [6,7]. Accordingly, our combined method includes four distinct steps (see Fig. 18), namely: (1) defining the research questions(s)/objective; (2) data collection (or searching the literature); (3) data extraction, organization, and coding; and (4) data analysis (combinations, correlations, and reporting). For the purpose of this article we will focus on the second and third steps.

*Data collection* commenced in June 2017. Due to the relative dearth in the peer-reviewed literature and inconsistency in the terminology used to describe stream daylighting, two decisions were taken early in the data collection process: to extend our literature search beyond peer reviewed sources to include grey literature and, by consequence, to scrutinize the sources’ contents to evaluate each source's latent and manifest content. This led us to characterize the sources’ types whether peer reviewed or grey literature and the latter's types as: books, book chapters, institutional reports, workshop proceedings, conference papers, and supervised students’ work. The quality of the grey literature sources was assessed through careful reading of each source's text and by reviewing the authors’ institutional affiliations and disciplinary expertise. More importantly, the initial careful reading of the collected sources revealed inconsistencies in the terminology used in the literature when referring to stream daylighting which led us to compile a list of these terms.

Accordingly, we expanded our search. We used two academic search engines Primo and Google Scholar, augmented by Google. Our primary data collection criterion was to identify all English-language sources that discuss stream daylighting with one exception for a report by the City of Zürich, which we translated from Swiss-German to English for inclusion in this study due to its insights into Zürich's unique policy-driven approach to stream daylighting. Therefore, for our iterative data collection process, we applied Boolean commands with the keywords: ‘stream’, ‘river’, ‘creek’, and ‘brook’ and combined them with one or more of the root words that we identified were in use to refer to stream daylighting, namely: ‘bury’; ‘deculvert’; ‘establish’; ‘naturalize’; ‘open’; ‘reclaim’; ‘recover’; ‘regenerate’; ‘rehabilitate’; ‘renovate’; ‘restore’; ‘resurface’; ‘revitalize’; ‘uncover’; and/or ‘unearth’. Concurrently with this Boolean search, the content analysis of the already identified sources enabled us to supplement our data collection in two ways. Firstly, by scrutinizing these sources’ lists of references for other sources that mention stream daylighting. And secondly, by including in our Boolean keywords the names of the stream daylighting case studies/projects mentioned in these sources, such as: the ‘Cheonggyecheon’ (the restored stream in Seoul, South Korea that is largely considered a notable example of reintroducing a lost stream to the urban landscape), ‘Zürich’ (the only city in the world with a policy dedicated to stream daylighting), and ‘Bani Hanifa’ (the only daylighting project in an arid context). Eventually, our purposeful methods yielded a total of 115 sources on stream daylighting, including peer-reviewed and grey literature.

*Data extraction, organization, and coding* took place concurrently with the data collection whereby due to our combined method, we applied four parallel processes: one for the manifest content and three for the latent content. Firstly, we recorded the sources’ manifest content and organized it within a Microsoft Excel document, including each source's: title, publication date, publication type and publication origin.

Secondly, we extracted the latent content from each source's text by thoroughly reading each source's content and compiling an annotated bibliography in a standardized format with information on: each source's dominant discussion themes and sub-themes, the stream daylighting case studies/projects mentioned in each source, each source's geographical scope, and each source's discussion of climate change adaptation and mitigation as it relates to stream daylighting (if any). The annotated bibliography's standardized format provided an objective and rule-guided format for recording and organizing (coding) data, hence, reduced the likelihood of researchers’ bias [6,8]. Thus, once a critical mass of sources had been reviewed (around 50 sources), we transferred the latent content from the annotated biography using open coding (i.e., content-relevant headings named according to content-characteristic words) to the same Microsoft Excel document as the manifest content (raw data). The coding of the latent data in the Microsoft Excel document converted the qualitative information into quantitative metrics (counts) [9]. Within this coding document, the higher-order themes and more detailed sub-themes were freely generated by sorting and collapsing similar content headings. Eventually, this process yielded an organizational hierarchy of nine dominant (higher-order) themes, namely: ‘daylighting’, ‘ecology’, ‘economy’, ‘hydrology’, ‘infrastructure’, ‘politics and policy’, ‘sociocultural’, ‘built form and urban design’, and ‘inclusive planning’. Under these nine themes, 53 second-order categories or sub-themes emerged. These themes and sub-themes were collectively exhaustive but not mutually exclusive –in other words, each source covered, at a minimum one theme and subtheme, but hypothetically, it may cover at a maximum all themes and subthemes. Table 1 provides a detailed description of the coding description for all the themes and sub-themes (sub-categories) that exist in the raw data of Dataset I (to view and/or download Dataset I, see [1]). We used Dataset I as a springboard for delving further into the literature, specifically, with regards to climate change and the stream daylighting case studies/projects as well as for creating the interactive Tableau Dashboard (Dataset IV).

Thirdly, in order to understand how the sources discussed stream daylighting in relation to climate change, we re-read all the sources that were coded under the ‘climate change’ theme. This second reading of the texts specifically sought, from each source's text, information on how each source connected stream daylighting to climate change whether as a nature-based solution for climate adaptation and/or mitigation, and whether stream daylighting's climate change impacts relate to the literature's remaining 52 subheadings (to view and/or download Dataset II, see [1]).

Lastly, we delved deeper into the stream daylighting case studies mentioned in the literature sources. One of this research project's objectives was to map the locations of stream daylighting examples around the globe. Accordingly, we created a separate Microsoft Excel document where we documented information on the case studies/projects mentioned in the literature sources, which led to Dataset III (to view and/or download this dataset, see [4]). We augmented this dataset with information gathered from other sources such as, newspaper articles, websites, Google Maps…etc. (see column T in this dataset). We used this Dataset III to create the Interactive Map (Dataset V).

Finally, our combined method capitalizes on the strengths of both systematic literature review and content analysis methodologies allowing us to gather and analyze all the English language literature sources on stream daylighting (systematic review) [10], while ensuring a rigorous qualitative and quantitative analysis of copious textual data, obtained from multiple sources over long time periods (content analysis) [11]. The combination facilitated an in-depth understanding of the multifaceted practice of stream daylighting that spans multiple disciplines [8,[11], [12], [13], [14].

## Ethics Statement

This research project was funded by the Social Sciences and Humanities Research Council of Canada (SSHRC) under the file name 435-2016-0243. SSHRC was not involved in the study design nor in the collection, analysis and interpretation of the data. SSHRC was not involved in the writing of this manuscript of the decision to submit it for publication.

## Declaration of Competing Interest

The authors declare no other competing financial interests or personal relationships which have, or could be perceived to have, had influence on the work reported in this article.

## Appendix Supplementary materials

Image, application 1

## Footnotes

1 The sample was reduced to exclude portions of the data deemed less significant, including projects identified as ‘n.d.’ (unknown whether they are channelized or naturalized). We also excluded the ‘symbolic’ projects due to the small sample size with available information (n = 1) and, of course, those which did not report a measurement for average length.

2 Projects identified as ‘n.d.’ (not defined: i.e., unknown whether they are channelized or naturalized) and those coded as ‘symbolic’ projects were excluded altogether from this analysis.

3 The latter recreated the historic watercourse within a publicly accessible space by pumping water from the Han River and from underground sources (Busquets 2011, Lee 2004).
